# The GIBACHT fellowship: a multilateral initiative for strengthening capacity in biosafety and biosecurity towards pandemic preparedness

**DOI:** 10.3389/fpubh.2024.1385579

**Published:** 2024-08-01

**Authors:** Eva Mertens, Marlow Zimmermann, Janine Dywicki, Min-Hi Lee, Joachim Pelikan, Barbara M. Bürkin, Elizeus Rutebemberwa, Axel Hoffmann

**Affiliations:** ^1^Bernhard Nocht Institute for Tropical Medicine (BNITM), Hamburg, Germany; ^2^Global Partnership Initiated Biosecurity Academia for Controlling Health Threats (GIBACHT), Hamburg, Germany; ^3^Robert Koch Institute (RKI), Berlin, Germany; ^4^Global Partnership Initiated Biosecurity Academia for Controlling Health Threats (GIBACHT), Berlin, Germany; ^5^Swiss Tropical and Public Health Institute (Swiss TPH), Basel, Switzerland; ^6^University of Basel, Basel, Switzerland; ^7^Global Partnership Initiated Biosecurity Academia for Controlling Health Threats (GIBACHT), Basel, Switzerland; ^8^African Field Epidemiology Network, Kampala, Uganda; ^9^Global Partnership Initiated Biosecurity Academia for Controlling Health Threats (GIBACHT), Kampala, Uganda

**Keywords:** biosafety, biosecurity, non-proliferation, capacity strengthening, training-of-trainers (ToT)

## Abstract

The German Biosecurity Programme was launched in 2013 with the aim to support partner countries overcome biological threats including natural outbreaks or the intentional misuse of highly pathogenic agents. As part of this programme, this paper describes the development and implementation of a multilateral biosafety and biosecurity training initiative, called ‘Global Partnership Initiated Biosecurity Academia for Controlling Health Threats’ (GIBACHT). To achieve its objectives, GIBACHT implemented a blended-learning approach with self-directed, distance-based learning phases and three training-of-trainer workshops. The programme follows Kirkpatrick’s model of learning to guarantee sustainable effects of improved knowledge and skills. One hundred nine fellows from 26 countries have been trained in seven cohorts. Many GIBACHT alumni have established additional biosafety/biosecurity trainings in their home countries. The knowledge exchange is strengthened by the implementation of a Moodle-based alumni network. GIBACHT has the potential to contribute to strengthening the capacities of partner countries in Africa, the Middle East, and South and Central Asia to respond and build resilience to biological threats.

## Introduction: background and rationale for the fellowship programme

Due to steadily increasing human mobility and international trade, infectious diseases can rapidly occur in multiple places, often distantly located from each other. New diseases emerge, often due to zoonotic pathogen transfer from animals into the human population with subsequent human to human transmission (1). The global community is experiencing the effects of such a disease with the SARS-CoV-2 pandemic, caused by a novel coronavirus first reported in Wuhan, China, in December 2019 (2, 3). Previously, the 2009 H1N1-pandemic, which led to a substantial increase of global mortality (4), indicated that the management of a pandemic requires enormous resources (5). Besides overburdening health systems worldwide, the current SARS-CoV-2 pandemic has led to excess mortality (6), while also resulting in global socio-economic losses (7) with impacts on mental health (8) and bearing the risk of widening the societal divide (9).

Apart from ‘natural’ disease outbreaks, there is also a relevant threat of intentional release of infectious agents, or of accidental release while handling or shipping. There have been only a few known instances of attacks with biological agents, the best known example being the 2001 anthrax attacks in the United States (10). The impact and likelihood of further intentional incidents are difficult to predict, but the ricin plot in Cologne in 2018 showed that the threat of a terror attack with biological agents is relevant (11). Just as nuclear and chemical weapons, bioweapons are weapons of mass destruction, but their recognition and detection are time consuming, which delays the opportunity for taking specific countermeasures.

The 2014 Ebola outbreak in West-Africa demonstrated how an infectious disease caused by a highly virulent pathogen can lead to an international crisis in countries with insufficiently prepared health systems, which required it to be tackled as an international cooperative effort (12). The lack of diagnostic capabilities (13), as well as well-trained personnel capable of implementing an outbreak response (14), have been critical in several countries. In Nigeria, a cadre of people with field epidemiology training was available and managed to contain the spread of the Ebola virus after it had been imported by an infected a businessman from Liberia in the summer of 2014 (15). While the global response to the Ebola outbreak was delayed, the epidemic response in Nigeria was immediate and effective limiting the epidemic to only 20 cases countrywide. To a large extent, this epidemic response consisted of case isolation and daily follow-up of all contacts for 21 days (16, 17).

### Learning environment

In 2013, the German Biosecurity Programme was launched by the German Federal Foreign Office under the auspices of the G7 Global Partnership against the Spread of Weapons and Materials of Mass Destruction. This framework programme aims to minimise risks associated with highly pathogenic agents and their potential misuse in partner countries through awareness raising, capacity strengthening, and scientific exchange.

The Global Partnership Initiated Biosecurity Academia for Controlling Health Threats (GIBACHT) is a multilateral one-year biosafety and biosecurity training programme within this framework. GIBACHT completed its seventh cohort in October 2021 and further cohorts are underway. It targets postgraduate public health professionals of partner countries in Africa, the Middle East, and South and Central Asia. The programme is implemented in partnership between the Bernhard Nocht Institute for Tropical Medicine (BNITM) in Hamburg/Germany, the Robert Koch Institute (RKI) in Berlin/Germany, the Swiss Tropical and Public Health Institute (Swiss TPH) in Allschwil/Switzerland, and the African Field Epidemiology Network (AFENET) in Kampala/Uganda.

GIBACHT aims to provide knowledge on intentional and unintentional biological incidents and their control. In order to strengthen national capacity in disaster management and preparedness, GIBACHT sensitises for biosafety and biosecurity issues.

The specific objectives of the programme are:

- To create awareness for the risks associated with biological agents able to cause disease outbreaks and mortality across state borders, and to train fellows to understand and recognise the differences between intentional and unintentional incidents,
- To provide education and training in control of infectious diseases and the prevention of biological proliferation risks for European, African and Asian experts in the area of epidemiology, biology, and medicine,
- To establish and sustain European/African/Asian cooperation in international proliferation and infection control,
- To train experts in epidemiology, biology, and medicine as multipliers and trainers in biosafety, biosecurity, and proliferation control,
- To establish sustainable structures for the continuing education in infectious disease epidemiology, biosafety and biosecurity, and proliferation control through training-of-trainer workshops and enhanced networking across the GIBACHT cohorts,

The training programme with a total learning investment time of 250 h follows a blended learning approach, combining self-administered, topic-specific eLearning modules, face-to-face workshops with interactive teaching, exercises, and distance-based group and individual work. In addition, GIBACHT offers its fellows a digital alumni platform to foster the future collaboration between fellows, facilitators, and teachers.

Within this setting, GIBACHT was able to adapt rapidly to the evolving COVID-19 pandemic in early spring 2020. After Germany went into its first lockdown and international travel was heavily restricted, it was impossible to implement the annual GIBACHT workshops in the locations of Hamburg, Berlin and Kampala as planned. Moreover, by selecting experts with an important role in outbreak management, the majority of the fellows were involved in the outbreak response to the SARS-CoV-2-virus in their home countries at high levels of the health system. Therefore, the teaching consortium decided to change the three face-to-face workshops of 2020 and 2021 into a virtual setting. Three major changes were implemented:

- Whereas the workshops were initially planned for five consecutive days at each site, the 5 days were spread over a period of 2 weeks to give the fellows the opportunity to fulfil their work duties.
- Many of the input lectures and exercises of the curriculum of each workshop were given as ‘pre-recorded’ lectures, so that the fellows could decide by themselves on the best time for their learning.
- The online time of the virtual classrooms was decreased to around 60% of the planned lecture times; meaning that synchronous learning time was decreased and more focus was put on the asynchronous learning time.

GIBACHT could build on the experience of Swiss TPH, which transferred many of their programmes and courses in global health into online or hybrid blended-learning offers in a short time. With a focus on flipped classroom concepts, it was possible to further strengthen this approach based on *Adult Learning* (18) principles. These imply a more problem-oriented role for the learners, but also more responsibility for their own success. This means that the programme takes advantage of the fellows’ experiences and focuses on their individual professional situation, emphasising the analysis of experiences. Following the suggestion of Manning (19), the role of the ‘teacher’ should be the one of a ‘facilitator,’ who engages the fellows rather than purely transmits knowledge. The assumption behind this is that the training programme will positively influence the behaviour of the fellows, supporting the implementation of the newly acquired knowledge and skills at their workplace.

**Figure 1:**
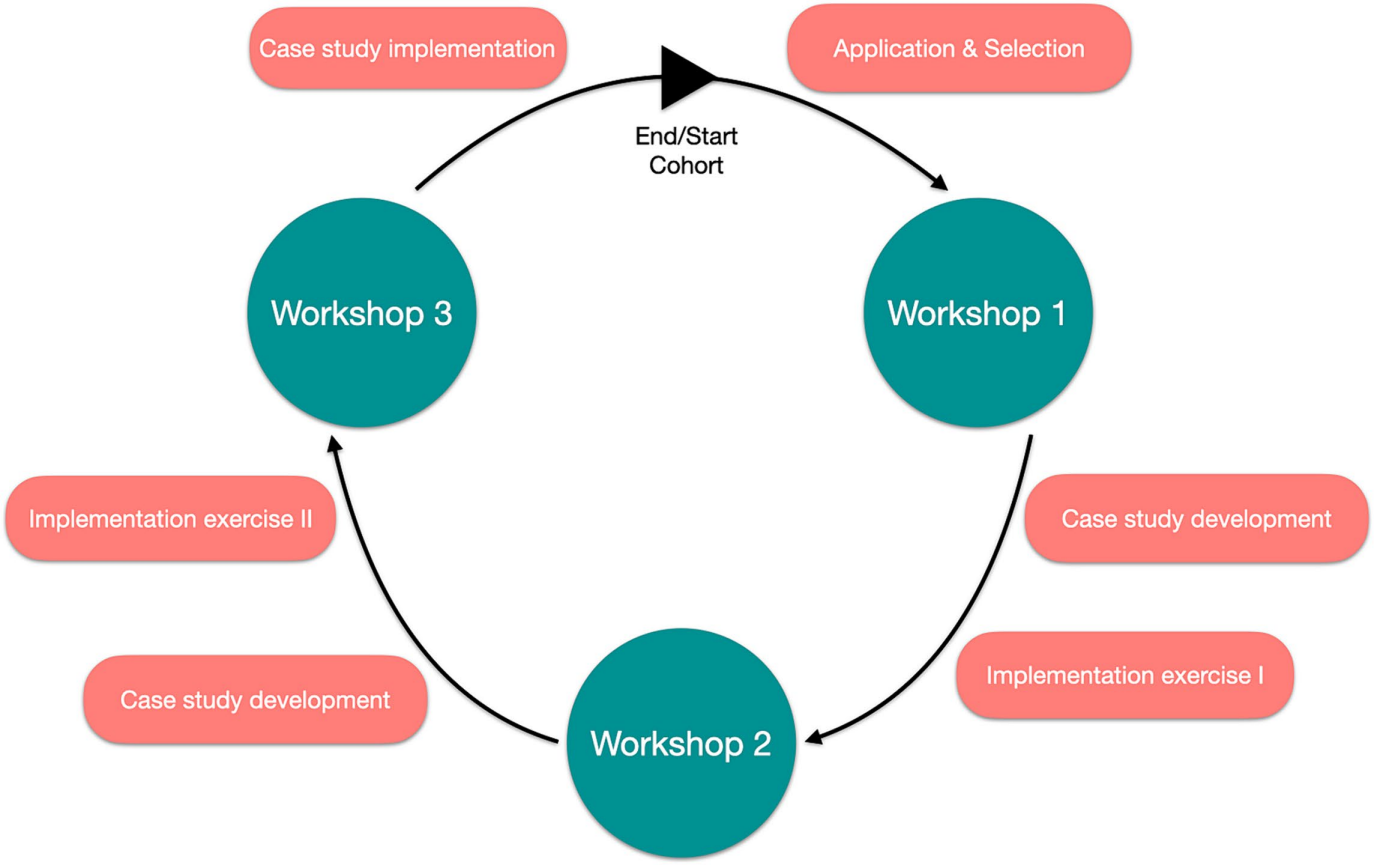
Structure of GIBACHT programme.

The structure of the GIBACHT curriculum itself has remained stable for the past seven cohorts from 2015 to 2019, including the online cohorts of 2020 and 2021 (Figure 1): Requirements to apply for the training are a degree in public health, medicine, or a life science, 2 years of professional experience in either public health or the medical sector, proficiency in English, and an endorsement of the employer to participate in the training. At the beginning of the programme, the fellows are working asynchronously through a set of introductory eLearning modules, which were created by expert authors from the partner institutions. The subject areas of the 20 modules include the topics biosafety and biosecurity, relevant biological agents (e.g., *Bacillus anthracis*, *Yersinia pestis*, and ricin), epidemiology, and public health. The didactical setup of these modules follows modern self-directed learning approaches (20). Fellows can work through the eLearning modules at their own pace, at a time that is appropriate for them, and are challenged by questions and quizzes in the course of the module.

The fellows meet for the first time during a five-day workshop at the BNITM in Hamburg/Germany. Although the fellows of the 2020 and 2021 cohorts could not physically join the workshops in Hamburg, Berlin and Kampala, as described above, the geographical workshop names were kept. During that period, the fellows receive in-depths lectures (in 2020 and 2021 additionally pre-recorded sessions) covering several topics (e.g., risk assessment, preparedness, crisis communication, non-proliferation, ethics) and an introduction to case study development. Practical exercises accompany the training, including group work sessions on outbreak investigation and packaging & shipping of biological samples. The teaching is adjusted to tender to adult learning techniques and is interactive in nature.

In between workshops, the fellows are working on the development, implementation and transfer of a biosafety/biosecurity-relevant case study to be used as teaching material in their home institutions and countries. This tabletop exercise usually consists of the presentation of either a real or fictitious biosafety/biosecurity scenario, which the target audience is working through by responding to a set of guided questions in group discussions. The development of the case studies is closely monitored and supervised by the GIBACHT consortium and GIBACHT alumni supporting the programme. This integration of GIBACHT alumni into current cohorts adds an additional layer to the training-of-trainers concept: it provides an additional facilitation experience in a professionally diverse and multi-national setting and applies biosafety and biosecurity content in the context-specific challenges conceptualised in the case studies. An integral part of the case study development is an implementation plan to use the case studies as a teaching tool at the home institutions of the fellows. This is in line with the overall ‘training-of-trainers concept’ of the programme.

During the second workshop, which takes place at the RKI in Berlin/Germany, the biosafety and biosecurity training is enriched by additional lectures, practical sessions, and real-time simulations of catastrophic health events. In group work, the fellows have to respond to fictional biological incidents from the perspective of a public health institution. This involves performing risk assessments of the evolving situation, deciding on control and containment measures, incorporating communication with the public, and developing preparedness plans for future incidents. One session is dedicated to an individual practical exercise on the use of Personal Protective Equipment (PPE).

The third GIBACHT workshop in Kampala/Uganda completes the one-year biosafety and biosecurity training. A main focus of the workshop is the pilot testing of the developed case studies with Public Health students invited from the Makerere University of Kampala. A pilot testing with students from Makerere University (Kampala, Uganda) was not possible in the virtual editions of 2020 and 2021. As the testing is an important feature of the training programme, this part was transferred to a virtual format with assistance of GIBACHT alumni. To prepare for this, the fellows receive training in the facilitation of case studies and how to positively interact with their target audience. The workshop also includes a field visit to farms in Uganda to visualise and explore the risk of pathogen transmission between wild animals and livestock as well as between animals and humans in a *One-Health* context.

At the end of the workshop, the fellows receive their certificates and are granted ten credit points according to the European Credit Transfer and Accumulation System (ECTS) points from the University of Basel.

Within the next 6 months after the final workshop, the fellows will apply the training-of-trainers concept and implement the case studies at their home institutions.

### Pedagogical framework underlying the fellowship programme

**Figure 2:**
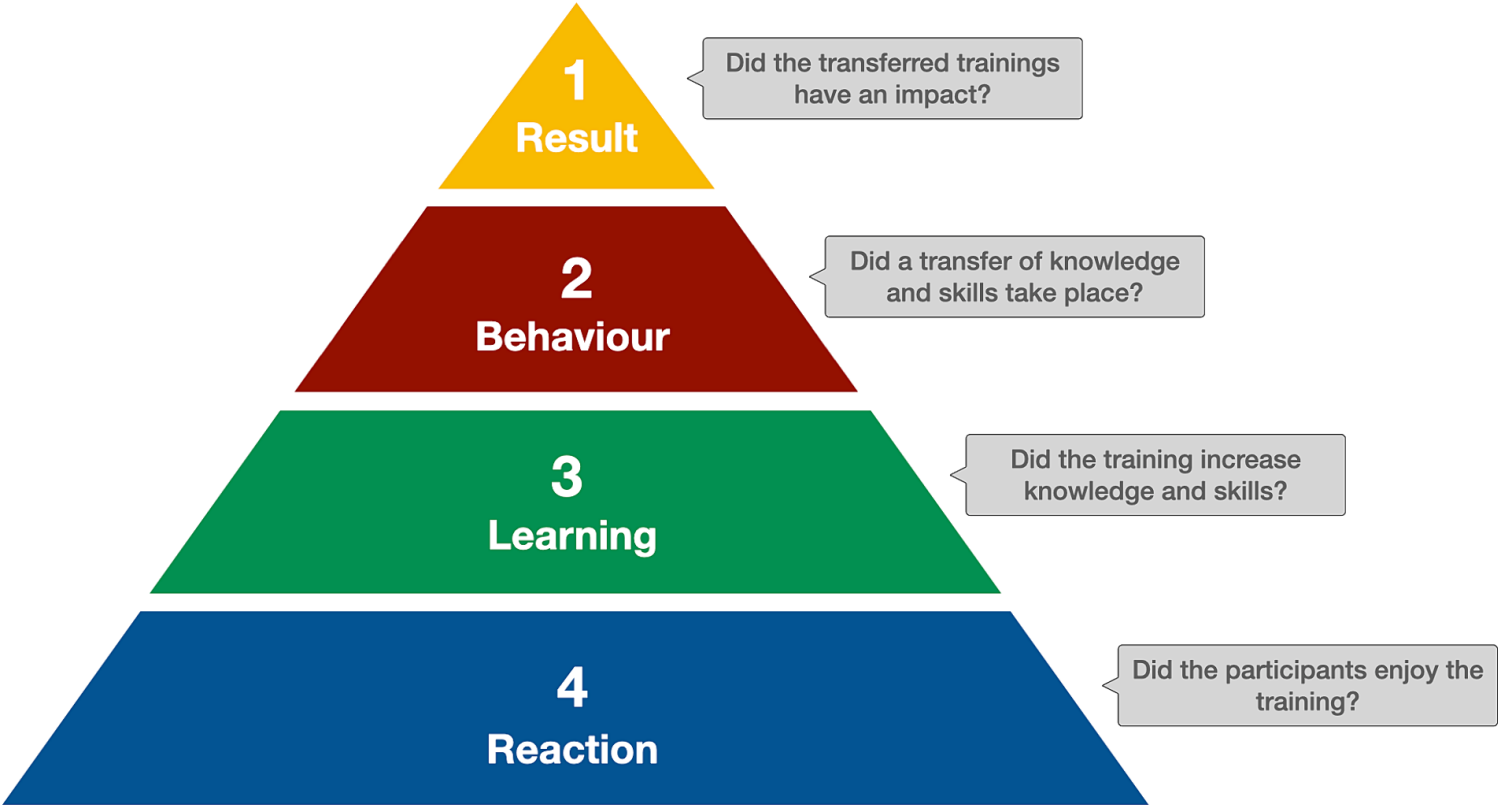
Kirkpatrick’s Model of training evaluations.

Kirkpatrick’s model (Figure 2) (21) was chosen as a framework to evaluate the training programme (22). The four levels of this framework are (a) Reaction: the initial reaction of participants to gain understanding of the training programme, (b) Learning: the effective absorption of information during the training and the relation to the learning objectives, (c) Behaviour: the influence of participants’ behaviour and the application of the new knowledge and skills on their job, and (d) Results: impact of the training at the participants’ institution/countries (21).

The level of *Reaction* was measured with individual interviews of fellows during the third workshop in Kampala. The interview contains standardised questions but also leaves room for comments on the training programme and its usefulness for the work of the fellows as well as their institution. In addition, all lectures, exercises and scenarios during the workshops are assessed by the fellows on the content and the educational approach using a standardised questionnaire at the end of each workshop day. The evaluation results have shown great acceptance of the programme with mean scores of 4.63 (SD = 0.18, *n* = 88) for content and 4.59 (SD = 0.2, *n* = 88) for educational approach on a 1–5 rating scale. Finally, the eLearning modules are evaluated by the fellows with a standardised questionnaire on their quality and usefulness for the individual learning.

The level of *Learning* focuses on the increase in knowledge and skills. In GIBACHT, several measures were introduced to assess – including formative assessments – the gain of newly acquired knowledge and skills. Prior to the first workshop, the fellows have to complete an online quiz covering the topics of the modules. On the first and the last day of each workshop, the fellows are asked to take a pre- and post-test, respectively, on selected topic areas covered during the workshop. In between the workshops, when fellows are working on a number of assignments, several feedback loops are introduced in which fellows get in contact with and receive support from facilitators and peers on their progress.

Many training evaluations stop at this second level – however, GIBACHT has the chance to measure also the level of *Behaviour* – the transfer of newly acquired knowledge and skills at the fellows’ workplace. Starting with the third cohort, fellows are required to plan and implement a number of trainings in their institution and document their experience in a short report. Although not objective in a narrow sense, these reports inform GIBACHT if achievements in transferring contents of the training programme to the fellows’ institutions were made, and which factors and/or conditions were supporting or hindering this process.

The level of *Results* is a long-term process, as the measurement has to be applied at least 1 year after fellows participated in the training. As the majority of former fellows are enrolled in GIBACHT’s digital alumni platform, it is planned to set up a survey to obtain at least basic information on such an impact.

### Assessment

**Figure 3:**
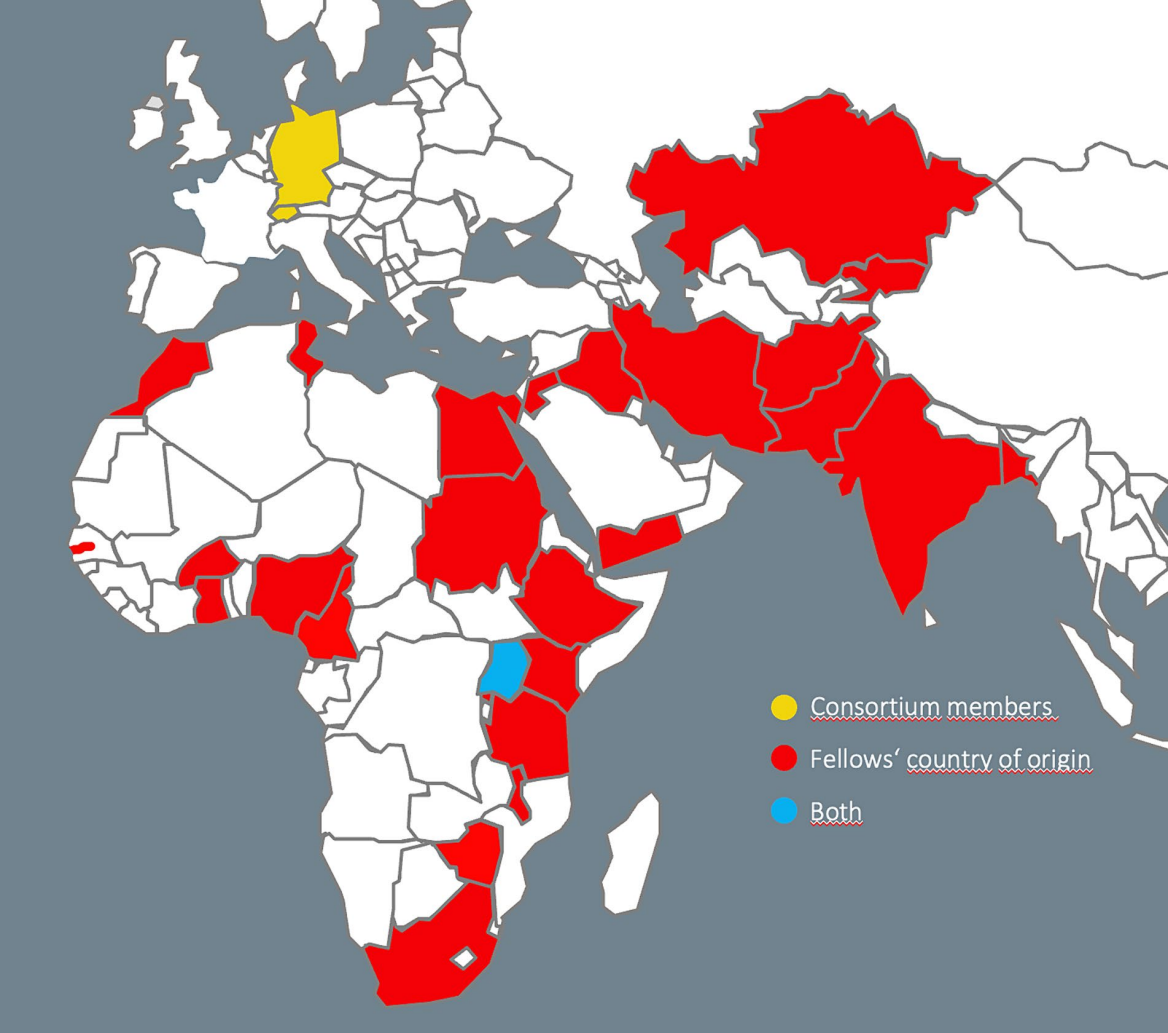
Countries of the GIBACHT consortium members are marked in yellow, GIBACHT fellows’ countries of origin are marked in red, Uganda (both) is marked in blue. Map template obtained from https://www.presentationmagazine.com.

GIBACHT is responding to the need of countries with insufficiently prepared health systems and risk of accidental and deliberate release of infectious agents. To date, 109 fellows from 26 countries (Figure 3) have been trained in seven cohorts, and 70 case studies have been developed. Many of the fellows have implemented the case study modules at their own institutions, shared their case studies with universitities as a teaching tool, or established biosafety/biosecurity trainings adjusted to the needs of their settings in their home countries, e.g., in Kyrgyzstan, Pakistan and Ghana. The knowledge exchange is further strengthened by the implementation of a Moodle (23) based alumni network and inclusion of GIBACHT alumni as workshop facilitators, case study reviewers and presenters at international conferences. In addition, the case studies developed by the fellows during the programme will be shared with the scientific community through publication in scientific journals.

The case study module was originally conceptualised as a means to reach the end of training trainers in biosecurity with a training material output. Feedback from GIBACHT participants and graduates suggests that it is being perceived as one of the outstanding features of the GIBACHT fellowship programme that was met with far more enthusiasm than anticipated. The process of the case study module and the conceptual framework of case study development in postgraduate education is described in a separate publication (Bürkin et al., in preparation).

## Discussion on the practical implications, objectives and lessons learned

GIBACHT as a multilateral programme for capacity strengthening in biosafety and biosecurity measures focused on sustainable improvement of knowledge and skills of health professionals working in this field. The training programme takes into account the variety of professional, cultural and religious backgrounds of its fellows and creates a platform of exchange that can enhance the learning outcomes after completion of the one-year fellowship period.

To achieve its objectives, GIBACHT implemented a blended-learning approach, with self-directed, distance-based learning phases and three 5-day workshops. Furthermore, the project takes into account the model of learning of Kirkpatrick (21) to guarantee sustainable effects of improved knowledge and skills.

Although these four levels are not clearly distinct, the attempt was made to measure the impact of each level to show success of the approach and to introduce possible changes in the curriculum, as described above.

On the lowest level, *Reaction*, the GIBACHT training approach clearly caters to the needs of the fellows and their employers – only short periods of absence from the workplace, combined with intensive phases of self-directed learning. Fellows appreciate the transfer of current knowledge in biosafety and biosecurity and the exchange with other fellows and facilitators. In general, the content of the taught sessions during the workshops and the quality of the facilitation (measured with the chosen educational approach for the sessions) were assessed on a very high level.

Changes in knowledge and skills are the focus of the second level *Learning*: Analysis of the pre- and post-test results indicates a clear knowledge increase for over 85% of fellows. In addition, the task of developing a case study in between the workshops and piloting it with their colleagues, as well as Public Health students during the Kampala workshop also shows the improvement of the fellows not only in technical - biosafety and biosecurity – knowledge and skills, but also in their ability to transfer this newly acquired knowledge. With this multiplier effect, GIBACHT contributes efficiently to a better implementation of the programme objectives in the home institutions and countries of the fellows.

At the *Behaviour/Application* level, GIBACHT demonstrates the advantage of the chosen blended-leaning approach: as participants have to implement a case study at their workplace and document it with a report, the GIBACHT consortium can give immediate feedback and steer the process of individual learning and its application by the fellow.

Showing new behaviour at the workplace is only partly dependent on individual factors, as important are the conditions at the workplace: are they supportive or hindering the fellow to apply their newly acquired knowledge and skills? These factors are important to measure the fourth level, *Impact*. This will be in the focus of a future study, based on an adaptation of the most-significant-change-technique (24). With this study, the sustainability of our training approach and the effects not only at the individual, but also the organisational level, will be demonstrated.

## Data availability statement

The raw data supporting the conclusions of this article will be made available by the authors, without undue reservation.

## Author contributions

EM: Writing – original draft, Writing – review & editing, Conceptualization, Funding acquisition, Project administration, Formal analysis, Visualization. MZ: Project administration, Writing – review & editing. JD: Writing – review & editing, Funding acquisition, Conceptualization. M-HL: Writing – review & editing, Conceptualization, Funding acquisition. JP: Writing – review & editing, Conceptualization, Funding acquisition. BB: Writing – review & editing, Conceptualization, Funding acquisition. ER: Writing – review & editing, Conceptualization, Funding acquisition. AH: Conceptualization, Methodology, Writing – original draft, Writing – review & editing, Funding acquisition.
